# The Function of BDNF and Its Receptor in the Male Genitourinary System and Its Potential Clinical Application

**DOI:** 10.3390/cimb45010008

**Published:** 2022-12-25

**Authors:** Xiaoli Tan, Liangyu Zhao, Yuxin Tang

**Affiliations:** 1 Department of Urology, The Fifth Affiliated Hospital, Sun Yat-Sen University, Zhuhai 510275, China; 2 Guangdong Provincial Key Laboratory of Biomedical Imaging, The Fifth Affiliated Hospital, Sun Yat-Sen University, Zhuhai 510275, China

**Keywords:** BDNF, prostate, testis, spermatogenesis, penis, erectile dysfunction, nerve regeneration, fibrosis, epididymis, vas deferens

## Abstract

**Background:** Brain-derived neurotrophic factor (BDNF), as a member of the nerve growth factor family, has been mentioned more and more frequently in recent literature reports. Among them, content about the male genitourinary system is also increasing. **Objective and Rationale:** BDNF plays an important role in the male genitourinary system. At the same time, the literature in this field is constantly increasing. Therefore, we systematically summarized the literature in order to more intuitively show the function of BDNF and its receptor in the male genitourinary system and its potential clinical application. **Search Methods:** An electronic search of, e.g., PubMed, scholar.google and Scopus, for articles relating to BDNF and its receptor in the male genitourinary system. **Outcomes:** In the male genitourinary system, BDNF and its receptors TrkB and p75 participate in a series of normal physiological activities, such as the maturation and morphogenesis of testes and epididymis and maintenance of isolated sperm motility. Similarly, an imbalance of the circulating concentration of BDNF also mediates the pathophysiological process of many diseases, such as prostate cancer, benign prostatic hyperplasia, male infertility, diabetes erectile dysfunction, penile sclerosis, and bladder fibrosis. As a consequence, we conclude that BDNF and its receptor are key regulatory proteins in the male genitourinary system, which can be used as potential therapeutic targets and markers for disease diagnosis.

## 1. Structure and Function of BDNF and Its Receptors

The BDNF monomer is a mature secretory polypeptide, composed of 119 amino acid residues, that contains three disulfide bonds and is an alkaline protein, mainly composed of a β-folded and irregularly curled secondary structure. It is highly expressed in the central nervous system of the brain, such as the amygdala, hypothalamus, and hippocampus, and it is one of the most abundant neurotrophic factors in the body.

**Figure 1:**
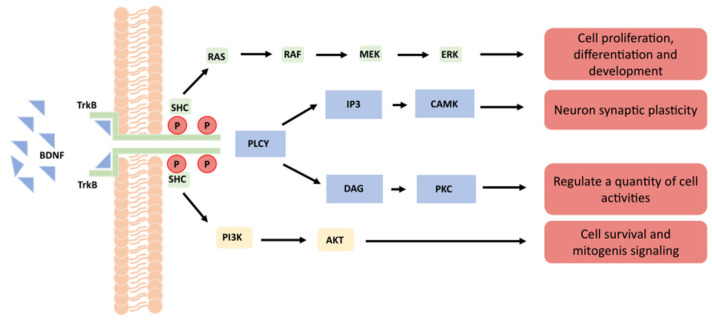
Schematic diagram of cell signal pathway after BDNF and TrkB binding.

BDNF acts by binding with two receptor systems—one being the high-affinity TrkB (tyrosine kinase B, also referred to as NTRK2) and the other being the low-affinity receptor p75 (a typical nonspecific receptor for all neurotrophins, also referred to as NGFR) [1] (Figure 1). After BDNF binds to TrkB, it activates the intracellular region, which leads to the enhancement of TrkB’s self-phosphorylation, thus activating RAS–MAPK and other pathways. The RAS–MAPK pathway leads to cell proliferation and differentiation, while the PLCγ pathway induces activation of inositol triphosphate (IP3), which further increases intracellular calcium release and ultimately leads to enhancement of the synaptic plasticity of neurons [2] (Figure 1). In addition, the latter pathway also allows survival of diacylglycerol (DAG) and regulates transcription factors, while the PI3K/Akt pathway plays an important role in cell signaling during mitosis. At the same time, this pathway can enhance hypoxia-inducible factor-1 (H1F1a) and increase the expression of TrkB. This regenerative feedback loop further enhances the positive effect of BDNF on cell division [3] (Figure 1).

By binding to p75, BDNF causes the intracellular kinase-binding domain to activate multiple signaling pathways and also regulates the dimerization of its receptor. The BDNF/p75 pathway plays a certain role in anti-cell proliferation, and the specific mechanism is detailed in the next chapter, “BDNF and the Prostate”.

mRNA expression in normal human tissues from GTEx, Illumina, BioGPS, and SAGE for the BDNF gene suggests that the gene encoding BDNF is transcribed in the human male prostate, testes, and bladder. Moreover, integrated proteomics about protein expression in normal tissues and cell lines from ProteomicsDB and MOPED for the BDNF gene shows that the BDNF protein is detectable in the prostate, urine, bladder, fetal testes, adult testes, and seminal vesicles of human males.

## 2. BDNF and the Prostate

The prostate gland, located behind the pubic symphysis and in the lesser pelvis, surrounds the root of the urethra. As the largest substantive organ in the accessory glands of the male genitalia, the prostate performs several physiological functions, including secreting prostatic fluid, participating in urination, assisting in ejaculation, and storing 5 α-reductase.

### 2.1. Physiological Function of BDNF in the Prostate

BDNF, together with its two receptors, are expressed in prostate blood vessels (macrophages), the glandular epithelium (fibroblasts), and interstitial cells (lymphocytes) [4]. Multiple research works have shown that BDNF may participate in the physiological activities of the prostate by paracrine and autocrine regulation at the same time [5]. BDNF binds to TrkB and activates multiple pathways, leading to a variety of biochemical reactions. TrkB mediates the proliferation and survival of prostate cells, as well as the upregulation of VEGF expression and angiogenesis. Furthermore, the combination of BDNF and p75 induces the programmed death of prostate cells. Other studies have shown that p75 retards cell cycle progression by inducing cell accumulation in the G0/G1 phase and reducing the S phase of the cell cycle. In addition, the death domain (Delta DD) of p75 can still play an anti-proliferation role toward cells in the absence of ligand dependence.

### 2.2. The Overexpression of TrkB and the Deletion of p75 Mediate the Pathophysiological Process of Prostate Cancer

Relevant experiments have shown that BDNF is highly expressed in prostate stromal cells and the androgen-responsive LNCaP prostate tumor cell line; on the contrary, it is not expressed in the androgen refractory TSU-pr1 prostate tumor cell line. These results indicate that the upregulation of BDNF autocrine expression gives rise to prostatic epithelial cells to get rid of their paracrine dependence on stromal cell-derived BDNF, which provides a prerequisite for the transformation of prostatic epithelial cells into androgen nonreactive pathology [5]. On the one hand, once prostate cancer cells can provide enough BDNF in an autocrine manner, the ability of tumor migration and diffusion is enhanced. On the other hand, the expression of long non-coding RNA (lncRNA) antisense BDNF (BDNF-AS) in prostate cancer cells is significantly downregulated. BDNF-AS can bind with the chromatin structure of the BDNF region to inhibit the transcription of BDNF, thereby reducing the secretion level of endogenous BNDF. The low expression of BDNF-AS in prostate cancer cells further enables cancer cells to obtain sufficient BDNF [6,7]. BDNF/TrkB has also been confirmed to be involved in epithelial–mesenchymal transition (EMT) and resistance to anoikis [8]. Interestingly, the downregulation of TrkB expression can promote the anoikis and partial reversal of EMT, while upregulation can cause the transformation of cell morphology similar to EMT. The specific mechanism remains to be studied. Overexpression of BDNF has also been detected not only in prostate cancer tissues, but also in benign prostatic hyperplasia (BPH) tissues, indicating that BDNF mainly contributes to stimulating cell proliferation [9].

The expression of the p75 receptor”In ’rostate cancer cells is just opposite to that of the TrkB receptor, which is gradually lost in malignant prostate tissue and not expressed in metastatic prostate cancer cell lines [4]. The deficiency of p75 removes the threat of programmed apoptosis from tumor cells. To sum up, the overexpression of TrkB and the deletion of p75 comprehensively guide the results of abnormal cell proliferation and form a tumorigenic environment.

## 3. BDNF and the Testes

The testes, consisting of spermatogenic tubules and surrounding connective tissue, are located in the scrotum, one on each side. Generally, the left testis is slightly lower than the right. In the connective tissue between the spermatogenic tubules, there are Leydig cells, which can synthesize testosterone. Spermatogenic cells at all levels are surrounded by the processes of Sertoli cells, which play the roles of supporting, protecting, and nutrition and are conducive to material transport and signal transmission between cells. 

The physiological function of the testes is mainly regulated by the “hypothalamic–pituitary–testicular axis”. Recent studies have found that BDNF can affect the physiological function of the testes by affecting this axis, as well as its own paracrine and autocrine.

### 3.1. BDNF Is Expressed by Sertoli and Promotes Proliferation of Leydig and Production of Testosterone

In previous studies, BDNF has been detected in the Sertoli and Leydig cells of human testes by immunocytochemistry and RT-PCR. Meanwhile, TrkB has been detected in spermatogonia, suggesting that BDNF may be involved in the paracrine regulation of spermatogenesis [10,11,12]. Collectively, BDNF is secreted by Sertoli cells, acts on spermatogonial stem cells, and guides a series of physiological activities through the BDNF/TrkB pathway. In a study by the same author, the expression of neurotrophic factor and its receptor in adult and fetal testes was detected [10], which suggests that BDNF may play a role in testicular maturation and morphogenesis [13]. On the one hand, it has been reported that BDNF can promote the proliferation of mouse TM3 Leydig cells in vitro, which may be induced by upregulating proliferating cell nuclear antigen (PCNA). On the other hand, BDNF upregulates the expression of 3b-hydroxysteroid dehydrogenase (Hsd3b1), steroidogenic acute regulatory protein (Star), and cytochrome P450 side-chain cleavage enzyme (Cyp11a1) through the ERK1/2 pathway, thus promoting the production of testosterone [14].

What is more, BDNF can also change the miRNA expression profile in TM3 Leydig cells (Normal mouse testicular Leydig cells). MiRNA regulates a large number of genes that perform different functions. BDNF may be mediated by miRNA to indirectly regulate the function of Leydig cells [15].

### 3.2. Physiological Function of BDNF in the Spermatozoa

Immunofluorescence staining showed that the BDNF protein is localized in three parts of human spermatozoa—the head, neck, and tail. The transcriptional and protein expression levels of the BDNF gene in oligoasthenozoospermic spermatozoa are lower than those of normal spermatozoa [16]. We hypothesize that the decreased expression of BDNF is related to the pathogenesis of some male infertility. There is evidence that BDNF has a protective effect on sperm oxidative stress (OS), which can maintain sperm activity and is very important for sperm to participate in sperm–egg fusion and subsequent fertilization in vitro. Under physiological conditions, sperm produce a small amount of reactive oxygen species (ROS). These small amounts of ROS are necessary to maintain sperm function. It has been reported that the scavenger in seminal plasma can remove high-concentration ROS. However, excessive production of ROS and/or deficiency of elimination leads to OS within sperm, which can lead to severe sperm damage. We hypothesize that BDNF may increase the activity of catalase by regulating antioxidant enzymes, such as increasing glutathione in cells and decreasing the activation of superoxide dismutase, so as to stabilize the concentration of ROS within a reasonable range, which prevents sperm OS [17,18]. Moreover, BDNF has also been proven to enhance the expression of sestrin2, a stress response gene involved in cell defense against OS, which is one of the mechanisms by which BDNF maintains sperm motility [15]. The exposure of BDNF causes the formation of a protein complex containing at least PKG-1 and p65/p50, which binds to the sestrin2 promoter, leading to the upregulation of its protein products and, ultimately, an anti-OS effect.

Above all, BDNF undertakes a large number of key tasks in the testes. We reasonably speculate that the absence or low secretion of BDNF will lead to a disorder of the paracrine regulation between Sertoli cells and spermatogonial stem cells and the mechanism of sperm defense against OS, leading to oligospermia or weak sperm, which may be the cause of some male infertility.

## 4. Overexpression of BDNF Mediates Bladder Fibrosis

The urinary bladder, a cystic structure composed of smooth muscle, is located in the pelvis. The bladder wall can be divided into three layers from inside to outside: The mucous membrane, muscular layer, and outer membrane. As a urine storage organ, the bladder contains abundant nerve cells to form a bladder pull receptor to produce the sensation to urinate, so that neurotrophic factors, including BDNF, are also expressed in the bladder.

Many literature works have reported that the expression level of BDNF increases in the case of inflammation or oxidative damage. Supplementation of exogenous BDNF significantly increases the production and deposition of the extracellular matrix (ECM), especially the activity of collagen-1 and collagen-3 (less fibronectin) and matrix metalloproteinases (MMP-2 and MMP-9) [19], which is one of the significant features of fibrosis. BDNF boosts the activation of microglia and astrocytes through the TrkB-p38/JNK signaling pathway and aggravates neuroinflammation and mechanical pain of cystitis [20]. The upregulation of neurotrophic factors and their receptors in the bladder mucosa leads to nerve hyperplasia, which, in turn, causes bladder pain and hypersensitivity. Previous histopathologic studies have shown increased full-thickness nerve hyperplasia and collagen deposition in the bladder wall in patients with cystitis, which supported previous speculation [21]. Chronic cystitis eventually progresses to bladder fibrosis.

## 5. BDNF and the Penis

The penis is mainly composed of two penile cavernous bodies and one urethral cavernous body, which are covered with anadesma and skin. The penis has the functions of sexual intercourse, urination, and ejaculation. In recent years, research on the penis has focused on erectile dysfunction (ED). The literature we searched also reported a relationship between BDNF and ED, and the progress of BDNF in the treatment of ED.

### 5.1. BDNF Participates in the Pathogenesis of ED

On the one hand, sensory and autonomic neurodegeneration are common types of diabetic neuropathy (DNP), which are highly related to erectile function. On the other hand, BDNF is also highly correlated with neurons, which provides valuable research directions for researchers. Existing studies have shown that the BDNF protein is detected in spongy tissue, and the expression of BDNF is downregulated in the penile corpus cavernosum of diabetic ED rats [22]. BDNF has been reported to promote the recovery of erectile function after nerve injury by activating neurite re-sprouting mediated by the JAK/STAT pathway. Integrated regulation of BDNF by binding to the p75 and TrkB receptors leads to the accelerated myelination of Schwann cells and promotes neuronal survival and neurite outgrowth [23,24]. As indicated above, the low expression of BDNF may be one of the culprits of DNP.

Penile erection, a nerve-regulated vascular activity, is comprehensively regulated by sympathetic and parasympathetic nerves. In addition, BDNF plays an important part in maintaining the growth and development of sympathetic and sensory nerves. While one of the complications of diabetes is DNP, and the degeneration of sympathetic and sensory nerves is an important component of DNP. As a result of selective neurodegeneration, the activity of nitric oxide synthase (nNOS) decreases, resulting in reduced NO production, resulting in impaired NO-induced relaxation in the corpus cavernosa of diabetic patients. Persistent hyperglycemia in patients with diabetes causes the nonenzymatic glycosylation of various proteins in the body and the formation of a large number of advanced glycation end products (AGEs). In the presence of a large number of AGEs, the relaxation of smooth muscle damages the cavernous body of the penis [25]. AGEs may cause the onset of ED in diabetes by producing oxygen free radicals. Oxygen free radicals can induce oxidative cell damage and inhibit NO, thus leading to a reduction of cGMP and impaired relaxation of cavernous smooth muscle, which ultimately affects the function of the cavernous body of the penis [26,27]. In addition, in relevant experiments, the increased content of smooth muscle in the cavernous body appears to be a manifestation of the diabetic pathological state. If so, an increase in muscle content reflects a change in smooth muscle from a normal contractile state to an abnormal synthetic state, leading to erectile dysfunction. If BDNF is supplemented, the contractile properties of cavernous smooth muscle can be maintained to a greater extent, which suggests that the absence of BDNF not only affects the function of the cavernous nerve, but also affects the function of cavernous muscle, thereby causing ED [28]. The occurrence of ED may also be a neuropsychological factor other than cavernous factors, which is related to BDNF. Relevant experiments have confirmed that the BDNF content in the plasma of patients with depression and other mental diseases is decreased, and the comorbidity rate of depression and ED is high [29,30]. Therefore, BDNF may also be involved in the pathogenesis of psychogenic ED.

### 5.2. BDNF Participates in the Pathogenesis of PD

**Figure 2:**
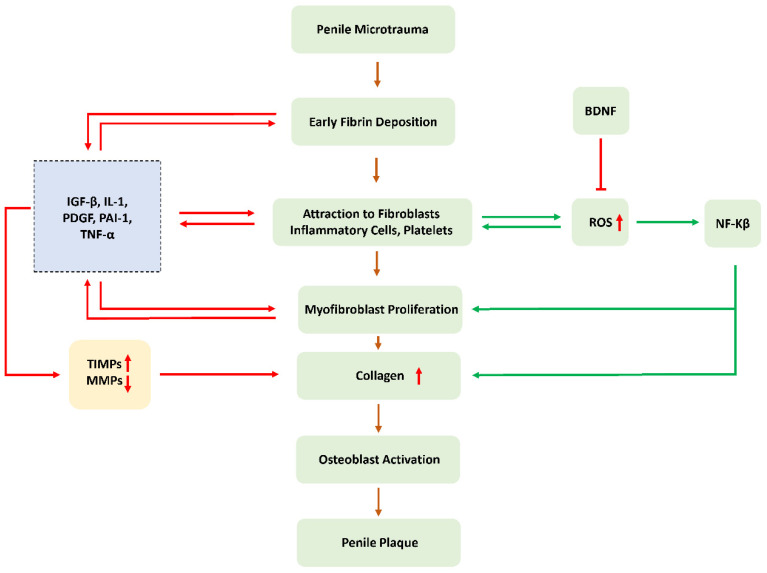
Schematic diagram of the effect of ROS on the progression of fibrosis.

As previously described, the regulatory effect of BDNF on ROS maintains ex vivo sperm motility, and there is increasing evidence that OS also plays an important role in the development of erectile dysfunction and subsequent Peyronie disease (PD) [31] (Figure 2).

**Figure 3:**
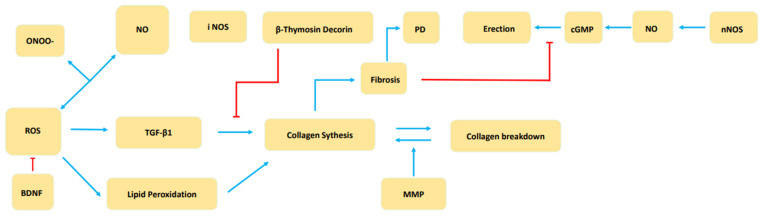
Schematic diagram of the balance between fibrosis and antifibrosis mechanisms in PD plaque.

Overproduction of ROS reduces the concentration of NO available for relaxing the smooth muscle of the cavernous body and may lead to long-term endothelial dysfunction. Repetitive stress and instability, which are more likely in ED patients, can lead to the development of PD. At the same time, excessive production of ROS further leads to nitro oxidative stress tissue damage and abnormal collagen deposition cycle, which is also a pathophysiological process of PD [32]. The balance between fibrosis and antifibrosis mechanisms in PD plaque is regulated by ROS as shown in (Figure 3):

ROS triggers the lipid peroxidation and synthesis of TGF-β1, two pro-fibrotic processes involved in atherosclerosis, arteriosclerosis, liver fibrosis, and other types of generalized inflammation and fibrosis. Therefore, we hypothesize that BDNF indirectly regulates the fibrotic process of PD plaque.

## 6. BDNF and the Reproductive Tract

For a long time, research on BDNF lay particular emphasis on the female reproductive tract. Therefore, there are few literature reports about the expression and function of BDNF in the human male reproductive tract. At present, articles related to BDNF and the male reproductive tract are only found in some animal models, and there are several references about BDNF in epididymis and vas deferens of rodents.

### 6.1. Physiological Function of BDNF in the Epididymis

BDNF and its receptors are expressed in specific cells in a growth-regulated fashion during epididymal development through specific polyclonal antibodies. In the early development stage of mouse epididymis, strong positive expression of p75 immunoreactivity has been found in the gonadal ridge in the mesenchyme of the mesenchyme, except the testicular cords. In the middle and later stages of epididymal development, most p75--positive interstitial cells begin to express truncated TrkB receptors in a developmentally regulated pattern. These findings suggest that BDNF and its receptors may play a regulatory role in the continuous growth and differentiation of epididymal cell types during epididymal development, but the specific mechanism remains to be studied. In the inner layer of the mesenchymal cells surrounding the developing epididymal duct, p75 and the expression of α- smooth muscle actin are negatively correlated. These results suggest that epididymal muscle cells may be differentiated from p75-positive cells [33]. BDNF expression has also been detected in epididymal fat tissue (EPF) of rat epididymal fat tissue (EPF) during the first two months of life, peaking at 20 days after birth and beginning to decline in adulthood. It is not clear how these secretory changes affect the development of epididymal adipocytes and the surrounding tissues. It is hypothesized that BDNF may participate in the maturation and/or morphogenetic events of postnatal adipose tissue development to regulate the manner of vascularization and/or sympathetic innervation during epididymal adipose tissue development [34,35]. In addition, BDNF not only has a potential role in the development of epididymal adipose tissue, but also plays a role in energy metabolism balance. Epididymal adipocytes only express the TrkB receptor, so BDNF regulates the dynamic balance of the energy metabolism only through the BDNF/TrkB axis [36]. Finally, in another study, the results showed that a decrease in BDNF in different dietary types is related to an increase in OS in the serum and epididymal adipose tissue [37,38]. In the absence of BDNF, in a high-sucrose and high-fat diet, the expression of antioxidant genes in epididymal adipose tissue is reduced and the level of inflammatory markers in type 2 diabetes patients is increased [39,40,41]. As mentioned above, BDNF plays an anti-OS role in the testes and sperm in various ways, which also plays an important role in epididymal adipose tissue.

### 6.2. Physiological Function of BDNF in the Vas Deferens

BDNF immunoreactivity is positive in the muscularis, subserosal ganglia, and nerves of the vas deferens of normal rats. After castration, BDNF immunoreactivity decreases in the vas deferens muscle, but not in the nerves. This indicates that BDNF plays a role in regulating the apoptosis of vas deferens cells under androgen deprivation. On the contrary, this shows that androgen dominates the expression of BDNF and its receptor in vas deferens muscle, which may indirectly affect the growth and development of the vas deferens muscle layer [42,43,44,45]. However, the specific role of BDNF in the vas deferens remains to be studied, and literature in this regard is lacking.

## 7. Potential Clinical Application of BDNF in the Male Genitourinary System

### 7.1. Treatment of BDNF in Male Genitourinary Diseases

In cavernous nerve injury (CNI) rats, intracallosal injection of BDNF can enhance the recovery of erectile function and promote the regeneration of nNOS-containing nerves [46]. BDNF can promote the recovery of erectile function after central nervous system injury by activating neurite re-germination mediated by the JAK/STAT pathway. However, the clinical application of this effect needs to be further explored and improved [47,48]. Subcutaneous injection of recombinant human methionyl brain-derived neurotrophic factor (rhBDNF) into the trunk skin improves sexual erectile function in diabetic ED patients [49]. With the rise in stem cell therapy, researchers have noticed that mesenchymal stem cells can also secrete various types of neurotrophic factors, including BDNF. This means that mesenchymal stem cells have the potential to treat diabetes-related ED. Injecting bone marrow mesenchymal stem cells (BM-MSCs) into sponges has been shown to improve erectile function and nerve regeneration in type 1 diabetic rats [50]. In addition, pelvic surgery, such as radical prostatectomy, easily results in iatrogenic damage to the cavernous nerve that provides the self-regulatory function of penile erection, which causes a high incidence of ED in patients after surgery [51,52,53]. Inspired by this, adipose-derived stem cells (ADSCs) infected with a lentiviral vector encoding rat BDNF (lenti-rBDNF) have been injected into the sponge, which has a promoting effect on the regeneration and functional recovery of cavernous nerves. The experiment proved that the therapeutic effect of BDNF combined with stem cells on nerve injured ED was better than that of single supplement of BDNF or single supplement of mesenchymal stem cells. However, the safety of lentivirus as BDNF introduced into ADSCs has not been confirmed in humans, and the experimental period was only four weeks. Whether the clinical effect is longer than this time is unknown. Another similar treatment plan is to use hydrogel and cell scaffolds coated with growth factor (including BDNF) to enhance the efficacy of ADSCs on ED, which can restore erectile function to a level close to normal [54].

Since BDNF and its TrkB receptors are also involved in the pathogenesis of prostate cancer, clinical research on drugs targeting this pathway and their interference with related carcinogenic effects is ongoing, including Cabozantinib, Larotrctinib, and Entrectinib. The success of early clinical trials of some drugs indicates that this pathway has the potential to become a new therapeutic target for advanced prostate cancer [55,56].

### 7.2. BDNF as a Potential Biomarker in the Male Genitourinary System

The content of the BDNF protein and the expression of BDNF mRNA in oligoasthenosperm are significantly lower than those in normal fertile spermatozoa, so BDNF could be used as a potential marker to indicate semen quality. More importantly, the expression profile of BDNF mRNA in oligoasthenosperm and normal fertile sperm is significantly different, which has value as a prognostic and diagnostic tool for fertilization [57,58,59].

As mentioned above, there are statistically significant differences in the expression of BDNF, TrkB, and p75 between normal prostate cells and prostate cancer cells, and the progression of prostate cancer is accompanied by an increase in TrkB signaling. Therefore, BDNF and its receptors have the potential to become tumor markers for the diagnosis of prostate cancer, as well as predictive markers of proliferation in the sense of tumor progression.

In the urine of BPH patients, the content of BDNF is also increased and has a gradient relationship with the severity of hyperplasia. Therefore, urinary BDNF can be used as a potential biomarker to evaluate lower urinary tract symptoms (LUTSs) in BPH patients [9].

### 7.3. Application of the Exogenous Supplement of BDNF and Agonists That Upregulate BDNF Expression

Because BDNF has a protective effect on the OS and apoptosis of isolated sperm, it is added to frozen or thawed semen to protect sperm from OS and apoptosis during freezing. It has clinical value in the field of reproductive medicine.

In addition, 4-methylcatechol (4-MC) has been reported to stimulate neurons to secrete BDNF. As an agonist for synthesis of BDNF, the previous research direction of this drug has focused on psychiatric system diseases, and its medicinal value in male urinary and reproductive systems still needs to be explored [60].

The BDNF protein has a short half-life and poor delivery in vivo. More importantly, exogenous supplemented BDNF not only binds to the TrkB receptor, but also to p75, a hybrid receptor that binds to a variety of neurotrophic factors to activate programmed cell death. For this purpose, researchers have designed a small-molecule TrkB receptor agonist similar to the BDNF structure—7,8-dihydroxyflavone [61]. This drug, which is currently being studied for psychiatric disorders and Alzheimer’s disease, has not been applied to the genitourinary system.

### 7.4. Antagonists for Downregulation of BDNF Expression

First, long non-coding RNA brain-derived neurotrophic factor antisense can complement the mRNA encoding BDNF to inhibit the translation process, which achieves the effect of blocking gene expression and downregulating the expression of BDNF. BDNF-AS has been found to be downregulated in tumor cells, which is associated with poor prognosis and short overall survival of prostate cancer patients. Therefore, the downregulation of BDNF-AS expression in prostate cancer patients has the potential to become a biomarker for predicting prognosis and poor survival. On the contrary, the upregulation of BDNF-AS expression may also be a new molecular intervention target for the treatment of prostate cancer.

Second, expression profile studies have also revealed the regulatory effect of miRNA (also known as microRNAs (MiR)) on the BDNF/TrkB pathway. miRNA can inhibit the translation of the BDNF/ TRKB pathway without being completely complementary to the target mRNA [62,63]. miRNAs antagonistic to BDNF expression, such as miR-101 and miR-107, have been studied for the targeted treatment of prostate cancer, and the previously mentioned drugs, such as Cabozantinib, Larotrctinib, and Entrctinib, have been developed.

Finally, a specific antagonist of the TrkB receptor, ANA-12, has been reported to have been developed. At present, it is only used for experimental purposes, such as the establishment of animal models and drug clinical trials [64,65].

## 8. Conclusions

To sum up, BDNF and its receptors, TrkB and p75, are widely expressed in the male genitourinary system and participate in a series of physiological activities. Second, an imbalance of the circulating concentration of BDNF also mediates the pathophysiological processes of many diseases. The correlation between BDNF and the pathophysiological processes of these diseases has been confirmed, but some specific mechanisms still need to be studied. Therefore, BDNF still has value of research in the pathophysiology of the male genitourinary diseases. Last but not least, BDNF has broad application prospects in the clinical application of the male genitourinary system. BDNF and its receptors still have potential value of research in the diagnosis and treatment of the male genitourinary system diseases. Many studies on clinical application remain in the stage of animal experiments and need to be further improved and demonstrated.

## Data Availability

The literature and data mentioned in this review are available on pubmed.

## Abbreviations

BDNF	Brain- derived neurotrophic factor
TrkB	Tyrosine kinase B
DAG	Diacylglycerol
H1F1a	Hypoxia inducible factor-1
Delta DD	The death domain
lncRNA	Long non-coding RNA
BDNF-AS	Antisense brain- de-rived neu-rotrophic factor
EMT	Epithelial-mesenchymal transition
BPH	Benign prostatic hyperplasia
PCNA	Proliferating cell nuclear antigen
Hsd3b1	3b-hydroxysteroid dehydrogenase
Star	Steroidogenic acute regulatory protein
Cyp11a1	Cytochrome P450 side-chain cleavage enzyme
TM3 Leydig cells	*Normal mouse testicular Leydig cells*
OS	Oxidative stress
ROS	Reactive oxygen species
ECM	Extracellular ma-trix
MMP	Matrix metalloproteinases
ED	Erectile dysfunction
DNP	Diabetic neuropathy
nNOS	nitric oxide synthase
AGEs	Advanced glycation end products
PD	Peyronie disease
TGF-β1	Transforming growth factor-β1
EPF	Epididymal fat tissue
rhBDNF	Recombinant human methionyl brain-derived neurotrophic factor
CNI	Cavernous nerve injury
BM-MSCs	Bone marrow mesenchymal stem cells
ADSCs	Adipose derived stem cells
lenti-rBDNF	Lentiviral vector encoding rat BDNF
LUTS	Lower urinary tract symptoms
4-MC	4-methylcatechol
MiR	MicroRNA
